# Aberrant RBMX expression is relevant for cancer prognosis and immunotherapy response

**DOI:** 10.18632/aging.205363

**Published:** 2024-01-11

**Authors:** Yilei Sheng, Kunjian Lei, Chengpeng Sun, Jia Liu, Zewei Tu, Xingen Zhu, Kai Huang

**Affiliations:** 1 Department of Neurosurgery, The Second Affiliated Hospital of Nanchang University, Nanchang 330006, Jiangxi, P.R. China; 2 The HuanKui Medical College of Nanchang University, Nanchang 330006, Jiangxi, P.R. China; 3 Jiangxi Key Laboratory of Neurological Tumors and Cerebrovascular Diseases, Nanchang 330006, Jiangxi, P.R. China; 4 Institute of Neuroscience, Nanchang University, Nanchang 330006, Jiangxi, P.R. China; 5 JXHC Key Laboratory of Neurological Medicine, Nanchang 330006, Jiangxi, P.R. China; 6 Department of Neuroscience, Yale School of Medicine, New Haven, CT 06511, USA

**Keywords:** RBMX, cancer prognosis, immunotherapy response, proliferation, invasion

## Abstract

Cancer accounts for the highest rates of morbidity and mortality worldwide. RNA binding motif protein X-linked (RBMX) is a nuclear RNA-binding protein, associated with certain types of cancer by participating in the integration of sister chromatids and a combination of ribonucleoprotein complexes. However, the specific role of RBMX in cancer immunity remains unknown. This study presents the aberrant expression levels, single-cell distributions, effective prognostic roles, immune cell infiltration associations, and immunotherapy responses of RBMX as a biomarker in various types of cancer. Moreover, it validates the aberrant expression of RBMX in clinical cancer samples. Furthermore, we also evaluated the relationships between RBMX expression and myeloid-derived suppressor cells in clinical samples by immunofluorescent staining. The results showed that knockdown of RBMX can impair the proliferation, migration, and invasion of liver cancer cells. Finally, we indicated that RBMX may play an immunoregulatory role in cancer progression, affecting the therapeutic effects of immune checkpoint inhibitors in patients with cancer.

## INTRODUCTION

Cancer is characterized by remarkably high rates of morbidity and mortality globally. In addition, it poses substantial burden on healthcare systems worldwide and negatively affects the economy [1]. Different types of genetic mutations have been found in patients with cancer; thus, a pan-cancer analysis of specific genes is urgently required to investigate the close correlations between the underlying molecular mechanisms and potential clinical prognosis [2, 3]. Despite the availability of increasingly effective therapy options for cancer, including surgery, radiotherapy, chemotherapy, and immunotherapy, the survival rates of patients with several types of cancer remain unimproved [4]. Immunotherapy has achieved great progress in the field of oncology, particularly against hematologic tumors. However, the effects of immunotherapy, such as immune checkpoints inhibitors (ICIs) and chimeric antigen receptor (CAR) T-cell therapy, on solid tumors remain unsatisfactory. Therefore, a pan-cancer analysis is necessary for the identification of novel biological targets and biomarkers involved in carcinogenesis, cancer progression, and immunotherapy response. Such knowledge would improve the precision of cancer therapy.

RNA-binding motif protein X-linked (RBMX) is a nuclear RNA-binding protein. It is the gene product of the X chromosome, maintaining genome stability during DNA splicing, DNA damage repair, and transcription [2, 5]. RBMX maintains the genomic stability by participating in the integration of sister chromatids and a combination of ribonucleoprotein complexes [6, 7]. It plays a crucial role in the regulation of cancer cell division and proliferation. Nonetheless, its role in cancer immunotherapy remains unclear. Recently, numerous research studies revealed the role of RBMX in cancer progression. For example, through network analysis of gene expression using abundant clinical data, Climente-Gonzalez et al. reported that RBMX mediated the essential drivers in tumorigenesis [8]. However, some studies revealed that RBMX may act as a tumor repressor. Mutations of RBMX found in patients with adenosquamous carcinoma suppressed the expression of RBMX, while tobacco-induced mutations of RBMX can increase the incidence of cancer among smokers [3, 9]. Moreover, Antonello et al. revealed the chromosomal abnormalities in vemurafenib resistance-related papillary thyroid carcinoma due to RBMX mutations [10]. However, a comprehensive pan-cancer analysis of the role of RBMX has not been conducted thus far, and its functions in cancer immunity have not been described.

In this study, we initially analyzed the transcriptional expression levels, genomic alterations, copy number variations, and DNA methylations of RBMX across different types of cancer using datasets from public databases. Subsequently, we verified the presence of aberrant RBMX protein levels in clinical liver hepatocellular carcinoma (LIHC), glioblastoma (GBM), and cholangiocarcinoma (CHOL) samples compared with adjacent tissues. Moreover, we applied single-cell RNA landscape analysis, prognostic analysis, and gene set enrichment analysis (GSEA) to unveil the potential clinical usage and explore the underlying biological functions of RBMX in different cancer types. Subsequently, we performed immune cell infiltration analysis/immune regulator correlation analysis and ICI response analysis to examine the cell infiltration associations and the predictive effect of RBMX in cancer immunotherapy response, respectively. Finally, by knocking down RBMX using short hairpin RNAs (shRNAs), we also validated the vital role of RBMX in maintaining the proliferative, migrative, and invasive capacities of liver cancer cells. The results revealed the significant efficiency of RBMX in predicting the immunotherapy response and providing a novel direction for the screening of effective prognostic biomarkers for cancer therapy.

## MATERIALS AND METHODS

### Data source

We acquired the pan-cancer tissue transcriptional data and human normal sample data from the TCGA pan-cancer dataset and GTEx datasets, respectively. These datasets were accessed from the UCSC Xena database (https://xenabrowser.net/datapages/). Moreover, the CNV data and methylation level of TSS data of 33 cancer types were also downloaded from the UCSC Xena database (https://xenabrowser.net/datapages/). Immunofluorescence data of MCF7 and U-2 OS cell lines were obtained from the Human Protein Atlas (https://www.proteinatlas.org/). The protein interaction information of RBMX was obtained from the ComPPI (http://comppi.linkgroup.hu) [11]. The abbreviations of different types of cancer are presented in Supplementary Table 1.

### Clinical sample collection

Seven GBM samples, three CHOL samples, and seven LIHC samples with paired adjacent normal tissues were collected. Prior to sample collection, informed consent was provided by all inpatients who underwent the resection operation in the Second Affiliated Hospital of Nanchang University (NCUSAH; Nanchang, China) from 2020 to 2022. Following excision, all tissues were immediately transferred and stored in liquid nitrogen.

### Single-cell analysis of RBMX

The TISCH web tool was utilized to perform single-cell analysis of RBMX (Gene) in the major lineage (Cell-type annotation) of all types of cancer (Cancer type). We examined the expression levels of RBMX in the pan-cancer setting, and quantificationally illustrated the data using scatter diagrams, violin plots, and a heatmap. The protocols for data acquisition, data processing, and cell annotation procedures were obtained from the TISCH website (http://tisch.comp-genomics.org/documentation/) [12].

### Prognostic analysis of RBMX in the pan-cancer setting

Four prognostic indices were included in our prognostic analysis, namely DFI, DSS, OS, and PFI. These follow-up data were downloaded from the UCSC Xena database (https://xenabrowser.net/datapages/). Univariate Cox proportional hazards regression and the Kaplan–Meier model were sequentially utilized to verify the essential role of RBMX in various types of cancer. Univariate Cox proportional hazards regression was applied to assess the importance of the continuous variable RBMX in predicting the prognosis of patients with cancer. Kaplan–Meier curve analysis was performed to assess the bivariate RBMX expression levels; the best cut-off value was selected using the “surv-cutpoint” function of the “survminer” R package (version 0.4.9).

### DEGs between the low- and high-RBMX subgroups

The samples for each TCGA cancer type were divided into low- and high-RBMX subgroups according to the RBMX mRNA expression levels. The top 30% and bottom 30% were defined as the high-RBMX subgroup and the low-RBMX subgroup, respectively. Differential expression analysis was conducted using the “limma” R package [13] to calculate the log2 (fold change) value and the adjusted p-value of each gene in different cancer types. Genes with adjusted p-values < 0.05 were defined as DEGs (Supplementary Table 2).

### GSEA

The “gmt” file of the hallmark gene set (h.all.v7.4.symbols.gmt) was downloaded from the Molecular Signature Database (MSigDB, https://www.gsea-msigdb.org/gsea/msigdb/index.jsp) [14], which contains a total of 50 hallmark gene sets. We used the “clusterProfiler” [15] package to perform the GSEA [16]. In addition, we calculated the normalized enrichment score and false discovery rate (FDR) for each hallmark gene in each cancer type using the DEGs obtained from the differential expression analysis.

### Immune cell infiltration analysis in TIMER2

The quantification of immune cell infiltration in different types of cancer was carried out using the TIMER platform. The associations of immune cell infiltration with RBMX expression in the pan-cancer setting were assessed using the TIMER2 database (http://timer.cistrome.org/) [17]. The associations between RBMX expression levels and 21 immune cell subsets (i.e., cancer-associated fibroblasts, CD4+ T cells, lymphoid progenitor cells, monocyte progenitor cells, endothelial cells, myeloid progenitor cells, hematopoietic stem cells, follicular helper T cells, eosinophils, γ/δ T cells, Treg cells, NK T cells, B cells, neutrophils, monocytes, dendritic cells, macrophages, NK cells, CD8+ T cells, and mast cells) were assessed by Spearman correlation analysis, and visualized in a assembled heatmap.

### Fluorescence immunohistochemistry of clinical samples

To gain insight into the correlation of RBMX with CD11b+ MDSC cell infiltration in LIHC, we performed fluorescence immunohistochemistry using three liver cancer samples obtained from the biological sample bank of the NCUSAH. We examined the correlation between the infiltration of CD11b+ MDSC and the expression levels of RBMX protein. The tumor tissues were embedded in paraffin and cut into slices (thickness: 5 μm) with a microtome. The slices were placed on glass slides and incubated in xylene solution for 15 min twice, 100% ethanol for 10 min, 85% and 75% ethanol for 5 min deparaffinization; the antigen was recovered in ethylenediaminetetraacetic acid buffer (pH 8.0). To block the antigen, the slices were incubated with a 3% bovine serum albumin solution for 30 min. Thereafter, the slices were incubated overnight with primary antibodies anti-CD11b (1:100) and anti-RBMX (1:100). Residual primary antibody was washed with phosphate-buffered saline (PBS) solution, and the slices were incubated with Cy3 (1:300) and AF488 (1:400) conjugated secondary antibodies for fluorescent labeling in the dark for 1 h. Finally, the nuclei were stained using 4’,6-diamidino-2-phenylindole dye for localization. The slides were observed and photographed with a fluorescence microscope.

### Immunotherapy prediction analysis

The statistical association between RBMX and familiar immunotherapy biomarkers (e.g., MSI, TMB, and other well-established immune checkpoint or regulator genes in different types of cancer) was evaluated through Spearman correlation analysis. Four immune checkpoint blockade therapy cohorts obtained from the Gene Expression Omnibus (https://www.ncbi.nlm.nih.gov/gds) were utilized to examine the predictive ability of RBMX regarding the response of cancer to immunotherapy.

The Gide2019 cohort included 32 patients with melanoma who received anti-CTLA4/anti-PD-1 therapy [18]; the Raiz2017 cohort included 26 patients with melanoma who received anti-PD-1 therapy [19]. In addition, the Lauss2017 cohort included 25 patients with melanoma who received adoptive T cell therapy [20]; and the Vanallen2015 cohort included 42 patients with melanoma treated with anti-CTLA4 therapy.

### Cell lines and cell culture

HCCLM3 and SK-HEP1 cell lines were purchased from the American Type Culture Collection (Manassas, VA, USA), and cultured using Dulbecco’s Modified Eagle’s medium supplemented with 10% fetal bovine serum (FBS; Gibco, USA) and 1 unit/ml penicillin and 1 mg/ml streptomycin (Gibco, USA) in 37° C incubator with 5% CO_2_ and 100% humidity.

### Plasmid constructs

Human RBMX targets (shRBMX-1: 5’-GGGCTTAATACGGAAACAAAT-3’, shRBMX-2: 5’-CCTCTCGTAGAGATGTTTATT-3’ shRBMX-3: 5’-CACCACCACCACGAGATTATA-3’) were designed and constructed by Service Company (Tianjin, China). The FV263 construct (U6-MCS-CMV-EGFP-hPGK-PuroR) was used to express the shRNAs. All plasmids were transfected using Lipofectamine 3000 Transfection Reagent (catalog number [Cat. No.] # L3000015; ThermoFisher, USA), and the transfected cells were used in subsequent cell assays after puromycin selection (2 μg/ml).

### Western blotting assay

The total protein of liver cancer cell lines was extracted using radioimmunoprecipitation assay lysis buffer (Cat. No. R0010; Solarbio, China), and the concentration of protein samples was quantified by a bicinchoninic acid kit (Cat. No. PC0020, Solarbio, China) on ice. Western blotting assay was conducted according to our previous publication [21]. The antibodies used were purchased from Proteintech Company (Wuhan, China), including RBMX2 polyclonal antibody (1:2,000 diluted, Cat. No. 17994-1-AP), glyceraldehyde-3-phosphate dehydrogenase (GAPDH) polyclonal antibody (1:5,000 diluted, Cat. No. 10494-1-AP), and horseradish peroxidase-conjugated Affinipure Goat Anti-Rabbit IgG(H+L) (1:5,000 diluted, Cat. No. SA00001-2).

### Cell proliferation assays

In the colony formation assay, we seeded 500 liver cancer cells from each group into each well of a six-well plate, together with 2 ml of complete medium; the culture medium was replaced once every 5 days for 3 weeks. After removing the culture medium, we fixed the cells with 4% formaldehyde for 10 min and stained them with 1% crystal violet for 30 min. Subsequently, the cell plates were rinsed with PBS five times. Finally, we photographed the wells and calculated the number of cell colonies in each well.

In the Cell Counting Kit-8 assay, liver cancer cells were seeded into a 96-well plate (2,000 cells per well in 100 μl of culture medium). Following the adherence of cells to the plate, we added 10 μl of CCK-8 reagent (Beyotime, Shanghai, China) to each well. Thereafter, the plate was placed in the incubator for 1 h to calculate the growth rate of cells. For this purpose, the absorbance in each well was measured at 450 nm wavelength, and the OD450 was recorded every 24 h to reflect the growth rates.

### Cell migration and invasion assays

Cells were seeded into a six-well plate. When the cells reached complete confluence, we removed the complete medium and performed a scratch using a sterile pipette tip. Next, we washed the six-well plate using PBS and added FBS-free medium for further culture of cells. Images of the scratch were captured at 0 h and 24 h.

Regarding the Transwell invasion assay, the pre-cool Matrigel matrix (Cat. No. 354263; Corning, USA) was equally spread in the upper chamber of 24-well Transwell plates. Next, the plates were incubated at 37° C for 2 h to solidify the Matrigel matrix. Subsequently, 20,000 liver cancer cells were seeded onto the solidified Matrigel matrix in FBS-free medium and co-cultured in the lower chamber with medium containing 10% FBS. After 12 h, invading cells that crossed the solidified Matrigel matrix and Transwell membrane were retained and stained with 1% crystal violet.

### Statistical analysis

The Wilcoxon rank-sum test was performed to compare RBMX expression between normal tissues and tumor tissues. A paired *t*-test was performed to calculate the statistical significance based on the RBMX immunohistochemistry score of paired clinical cancer and adjacent tissues. The Kaplan–Meier method and univariate Cox proportional hazards regression analysis were used to evaluate the prognostic value of RBMX expression in various types of cancer. Spearman correlation analysis was conducted to determine the correlation between RBMX and relevant factors. A chi-squared test was performed to calculate the responding ratio of an ICI-therapy responder and a non-responder between the low- and high-RBMX subgroups.

### Data availability statement

The original data used in this project can be downloaded from the UCSC (https://xenabrowser.net/datapages/) website.

## RESULTS

### Comprehensive landscape of RBMX in different types of cancer

**Figure 1:**
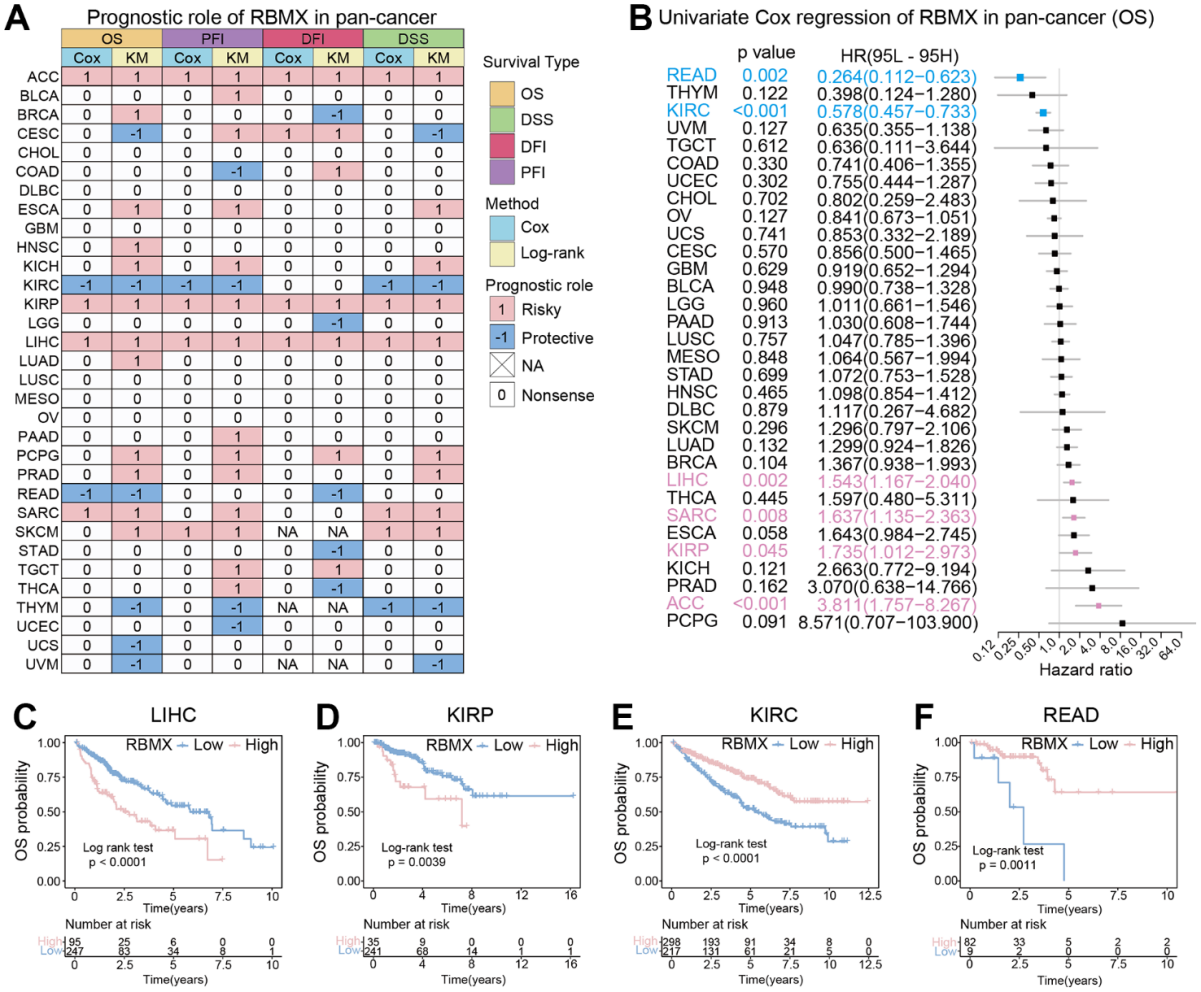
**Comprehensive landscape of RBMX.** (**A**) Transcriptional expression levels of RBMX in cancer based on TCGA and GTEx cohorts. (**B**) Different expression levels of RBMX between CHOL and normal cholecyst tissues. (**C**) Analysis of RBMX alteration frequency in different types of cancer using data from the cBioPortal database. (**D**) Pearson correlations between RBMX expression and RBMX copy number variation in each type of cancer. (**E**) Pearson correlations between RBMX expression and RBMX transcriptional start site (TSS) methylation in each type of cancer. (**F**) Immunofluorescence images of RBMX protein, nucleus, endoplasmic reticulum (ER), microtubules, and merged images in MCF7 and U2-OS cell lines. (**G**) The protein–protein interaction (PPI) network displayed interaction between the proteins and RBMX. (**H**–**M**) RBMX expression between clinical tumor samples and related normal samples in CHOL (**H**, **I**), LIHC (**J**, **K**), and GBM (**L**, **M**). CHOL, cholangiocarcinoma; GBM, glioblastoma; GTEx, Genotype-Tissue Expression; LIHC, liver hepatocellular carcinoma; RBMX, RNA binding motif protein X-linked; TCGA, The Cancer Genome Atlas.

The workflow was included in the Figure Abstract to better understand the content of this study. Initially, we sought to determine the role of RBMX in various types of cancer. Thus, we extracted data from The Cancer Genome Atlas (TCGA) and Genotype-Tissue Expression (GTEx) databases, and compared the RBMX transcriptional expression levels between the tumor and normal samples. Higher RBMX expression was found in 13 cancer types, including colon adenocarcinoma (COAD), CHOL, GBM, esophageal carcinoma (ESCA), kidney renal clear cell carcinoma (KIRC), head and neck squamous cell carcinoma (HNSC), etc. In contrast, RBMX expression was downregulated in acute myeloid leukemia (LAML), ovarian serous cystadenocarcinoma (OV), skin cutaneous melanoma (SKCM), thyroid carcinoma (THCA), uterine corpus endometrial carcinoma (UCEC), uterine carcinosarcoma (UCS), etc. The expression levels of RBMX were almost equal in breast invasive carcinoma (BRCA) and kidney renal papillary cell carcinoma (KIRP) (Figure 1A). Across all cancer types, RBMX expression was most significantly upregulated in CHOL compared with normal tissues (Figure 1B). Next, we applied genomic alteration analysis. We detected the highest rate of alteration of RBMX in UCS, with > 6% of patients carrying mutations as well as deep deletion (Figure 1C). Furthermore, we analyzed the correlations between copy number variation (CNV) and transcriptional expression in various types of cancer. The most remarkable correlation was observed in thymoma (THYM) (Figure 1D). Analysis of transcription start site (TSS) methylation showed a significant correlation between the TSS methylation level and RBMX expression in THCA (Figure 1E). Nevertheless, immunofluorescence images obtained from the Human Protein Atlas revealed that the RBMX protein was initially localized in the nucleus of the MCF7 and U-2 OS tumor cell lines (Figure 1F). Finally, we constructed a protein–protein interaction (PPI) network using sample data extracted from the compartmentalized PPI database (ComPPI website). The analysis illustrated the subcellular localization of proteins that closely interacted with RBMX. These proteins were distributed in the mitochondria, nucleus, cytosol, secretory pathway, extracellular space, and membrane (Figure 1G). Our analysis of immunohistochemical specimens also validated that RBMX protein was overexpressed in CHOL (n = 3), LIHC (n = 7), and GBM (n = 7) tissues compared with their adjacent tissues (Figure 1H–1M).

**Figure 8:**
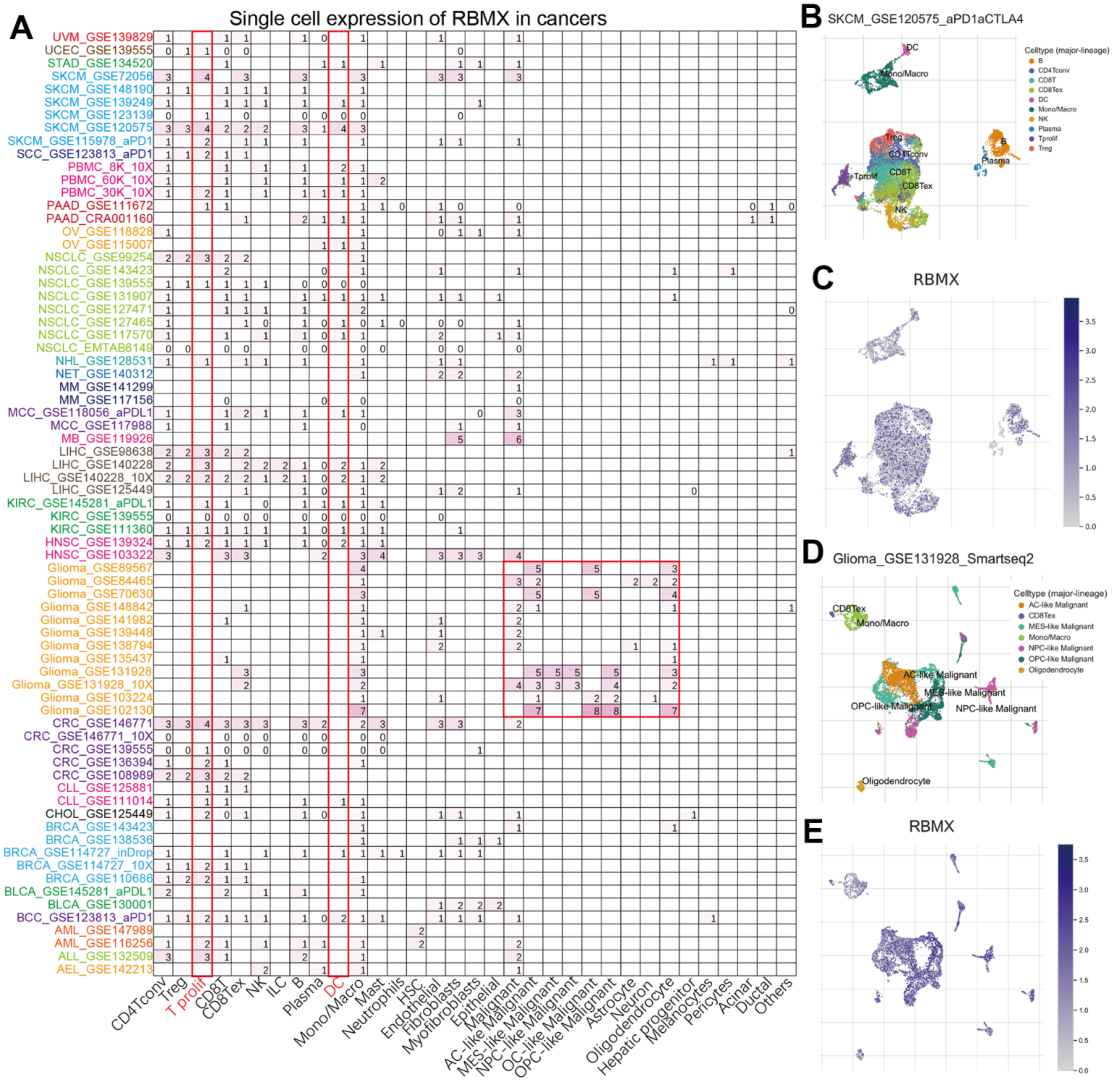
Figure Abstract. The workflow of our study.

### Single-cell analysis of RBMX in the pan-cancer setting

**Figure 2:**
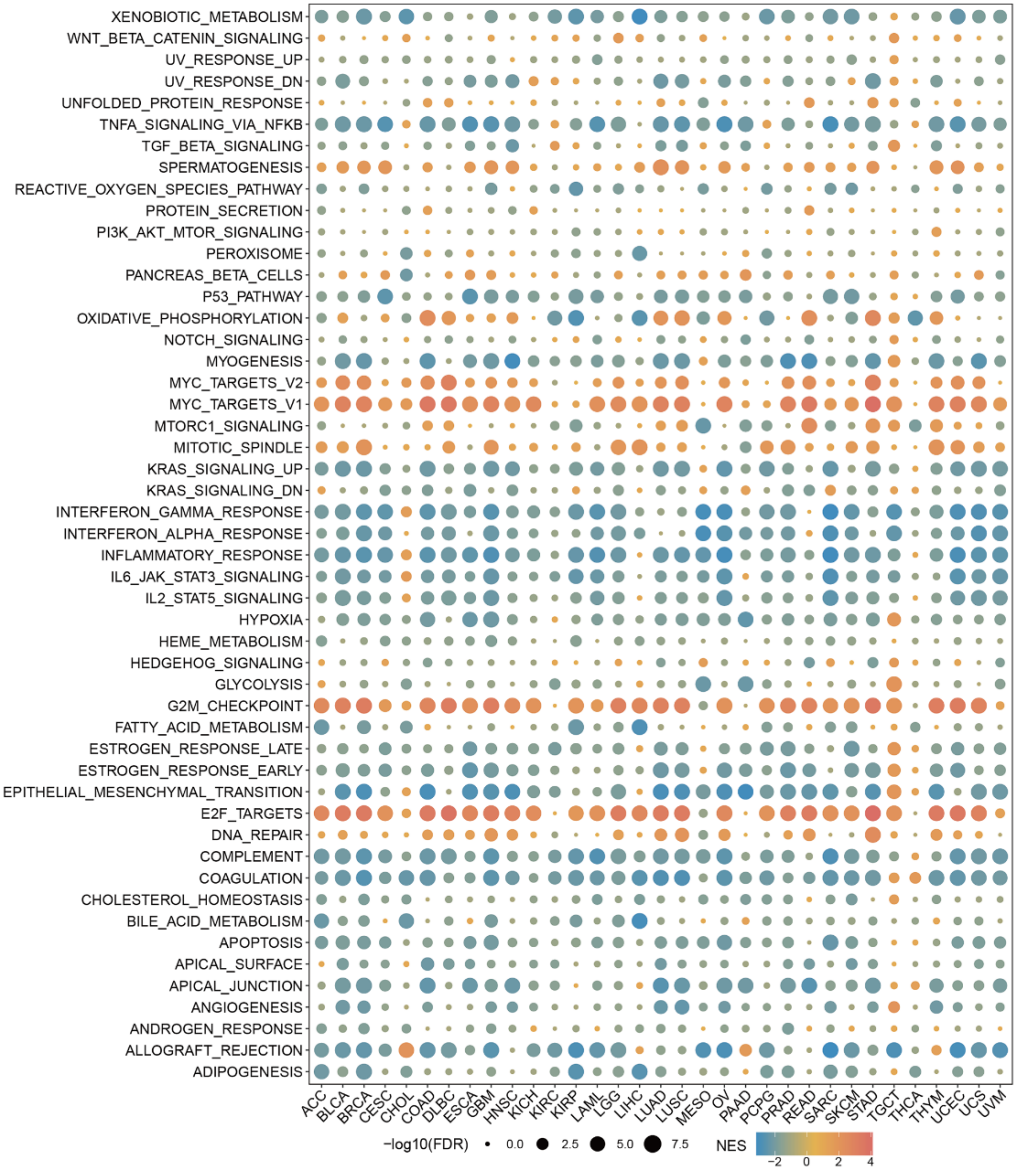
**Single-cell analysis of RBMX in different types of cancer.** (**A**) Heatmap exhibiting a comprehensive landscape of RBMX expression in 33 cell types based on 28 single-cell datasets (the number indicates the expression levels of RBMX). (**B**, **C**) Distribution of various cells in SKCM based on the GSE120575 database. (**D**, **E**) Distribution of various cells in glioma based on the GSE131928 database. RBMX, RNA binding motif protein X-linked; SKCM, skin cutaneous melanoma.

To further analyze the distribution of RBMX-expressing cells in tumor microenvironments, we applied the single-cell analysis of RBMX using the Tumor Immune Single-cell Hub (TISCH) webtool. The heatmap illustrated that the immune cells (particularly dendritic cells [DC] and T prolif) accounted for most RBMX expression in the majority of tumor types (Figure 2A). Moreover, we examined the GSE120575 dataset containing 16,291 cells from 32 patients with metastatic SKCM who received treatment with ICIs. The results revealed that RBMX was widely expressed in the plasma and immune cells, including DC, monocytes/macrophages, T prolif, CD8T, CD8Tex, CD4Tconv, regulatory T (Treg), natural killer (NK), and B cells (particularly in T prolif cells) (Figure 2B, 2C). Next, we analyzed the distribution of RBMX expression in the glioma microenvironment using the data of GSE131928, which includes 7,930 cells from 28 patients with glioma. The analysis indicated that RBMX was highly expressed in malignant cells and monocytes/macrophages (Figure 2D, 2E). These results revealed the abnormal expression of RBMX in different types of infiltrating immune cells. Therefore, higher RBMX expression may be involved in the immune defense, immune surveillance, and immune clearance of tumor cells in the tumor microenvironment, thus affecting tumor progression.

### Prognostic analysis of RBMX in the pan-cancer setting

**Figure 3:**
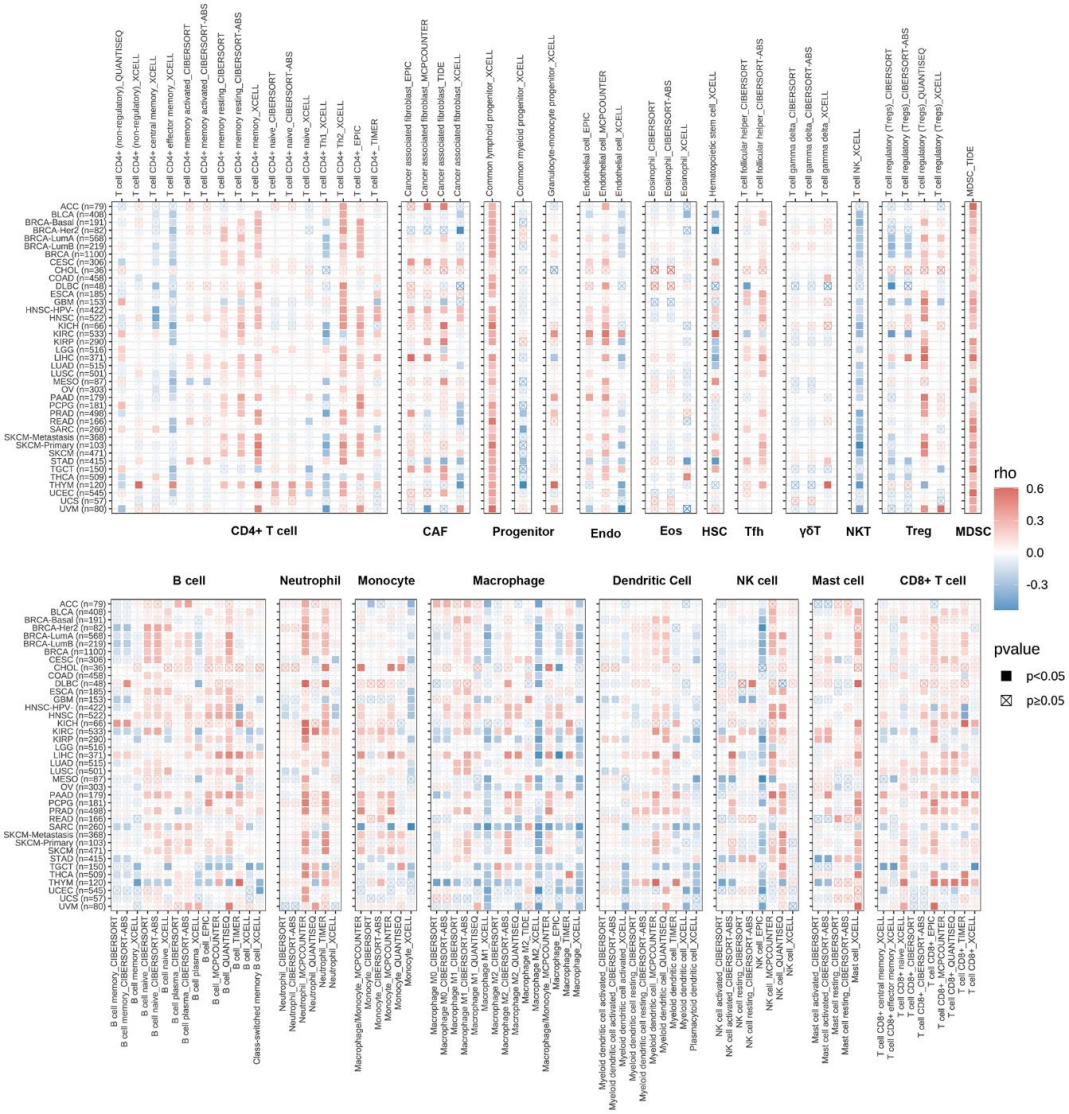
**Prognostic analysis of RBMX in the pan-cancer setting.** (**A**) Heatmap based on the univariate Cox proportional hazards regression and Kaplan–Meier models. The heatmap summarizes the correlation between RBMX expression and disease-specific survival (DSS), overall survival (OS), progression-free interval (PFI), and disease-free interval (DFI). Red and blue represent the risk and protective roles, respectively, in the prognosis of cancer. (**B**) Prognostic role of RBMX in the pan-cancer setting, illustrated by a forest plot based on the univariate Cox proportional hazards regression method. Red indicates the types of cancer for which RBMX was identified as significant risk factor. (**C**–**F**) Kaplan–Meier analysis indicated that higher RBMX expression was associated with worse clinical prognosis in LIHC (**C**) and KIRP (**D**). However, higher RBMX expression was predictive of better prognosis in KIRC (**E**) and READ (**F**). KIRC, kidney renal clear cell carcinoma; KIRP, kidney renal papillary cell carcinoma; LIHC, liver hepatocellular carcinoma; RBMX, RNA binding motif protein X-linked; READ, rectum adenocarcinoma.

Initially, we performed Kaplan–Meier and univariate Cox proportional hazards regression analyses in each cancer type to further explore the clinical application potential of RBMX in the prediction of cancer prognosis. The patients with low- and high-RBMX expression in the Kaplan–Meier survival analysis were divided into groups using the “surv-cutpoint” function of the “survminer” R package (0.4.9) to obtain the most significant cut-off value. The results were visualized in a heatmap (Figure 3A), showing the high relevance of RBMX and its prognostic value in the majority of cancer types. Overall survival (OS) analysis revealed that RBMX was a protective factor in patients suffering from cervical squamous cell carcinoma and endocervical adenocarcinoma (CESC), KIRC, rectum adenocarcinoma (READ), THYM, UCS, and uveal melanoma (UVM). In contrast, it was identified as a risk factor in BRCA, ESCA, kidney chromophobe (KICH), HNSC, KIRP, lung adenocarcinoma (LUAD), LIHC, prostate adenocarcinoma (PRAD), sarcoma (SARC), pheochromocytoma and paraganglioma (PCPG), and SKCM. Subsequently, we performed progression-free interval (PFI) analysis to evaluate the role of RBMX in tumor death, recurrence, and metastasis. The findings indicated that RBMX was a protective factor in COAD, KIRC, THYM, and UCEC. Moreover, the data obtained from the disease-free interval (DFI) analysis suggested that RBMX is a protective factor in BRCA, brain lower grade glioma (LGG), READ, stomach adenocarcinoma (STAD), and THCA. The disease-specific survival (DSS) analysis indicated such as role for RBMX in CESC, KIRC, THYM, and UVM. Nevertheless, RBMX was mostly recognized as a risk factor. Notably, RBMX was identified as a high-risk factor in adrenocortical carcinoma (ACC), KIRP, and LIHC, while it played a protective role in KIRC and THYM. We performed univariate Cox proportional hazards regression to further analyze the relationship between RBMX and the prognosis of patients with 32 types of cancer. The forest plot revealed that the expression of RBMX was upregulated and the OS time was prolonged in READ (hazard ratio [HR]: 0.264, 95% confidence interval [CI]: 0.112–0.623, p = 0.002) and KIRC (HR: 0.578, 95% CI: 0.457–0.733, p < 0.01). This tendency was reversed in LIHC (HR: 1.543, 95% CI: 1.167–2.040, p = 0.002), SARC (HR: 1.637, 95% CI: 1.153–2.363, p = 0.008), KIRP (HR: 1.735, 95% CI: 1.102–2.973, p = 0.045), and ACC (HR: 3.811, 95% CI: 1.757–8.267, p <0.001) (Figure 3B). The Kaplan–Meier survival analysis based on OS data showed that patients with different types of cancer could be classified into two subgroups with distinct prognoses based on RBMX expression. For example, higher RBMX expression was associated with poorer prognosis for patients with LIHC and KIRP (Figure 3C, 3D), whereas it was linked to better prognosis for patients with KIRC and READ (Figure 3E, 3F).

### GSEA of RBMX in the pan-cancer setting

**Figure 4:**
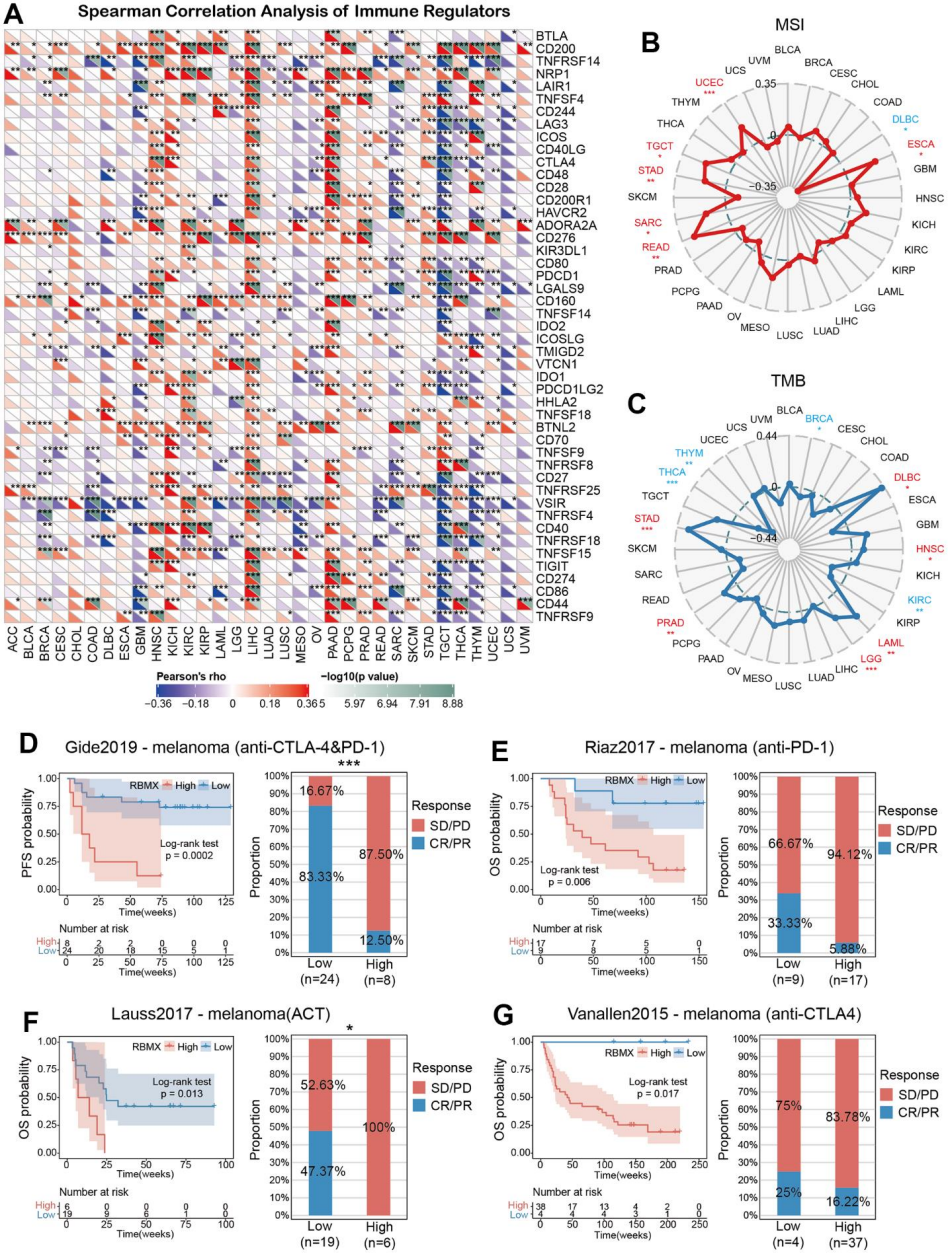
**GSEA of RBMX in the pan-cancer setting.** Heatmap illustrating the results of the GSEA for RBMX in the pan-cancer setting. The circle size indicates the enrichment of the false discovery rate (FDR) value in the pan-cancer setting. Red represents an increase in the normalized enrichment score (NES) in the pan-cancer setting; blue indicates the reverse. GSEA, gene set enrichment analysis; RBMX, RNA binding motif protein X-linked.

We sought to gain insight into the role of RBMX in the biological processes of various types of cancer. Therefore, we conducted GSEA to distinguish the cancer hallmarks of RBMX based on differentially expressed genes (DEGs) between the low- and high-RBMX subgroups in 33 types of cancer. The results indicated a significant correlation between RBMX expression and immune-related pathways, particularly tumor necrosis factor alpha (TNFA) signaling via nuclear factor kappa B (NFKB), interferon gamma (IFNG) response, interferon alpha (IFNA) response, and inflammatory in bladder urothelial carcinoma, COAD, diffuse large B-cell lymphoma (DLBC), lung squamous cell carcinoma, and STAD. Based on this evidence, RBMX may affect the tumor microenvironment by regulating the interaction between malignant tumor cells and immune cells (Figure 4).

### Immune cell infiltration analysis

**Figure 5:**
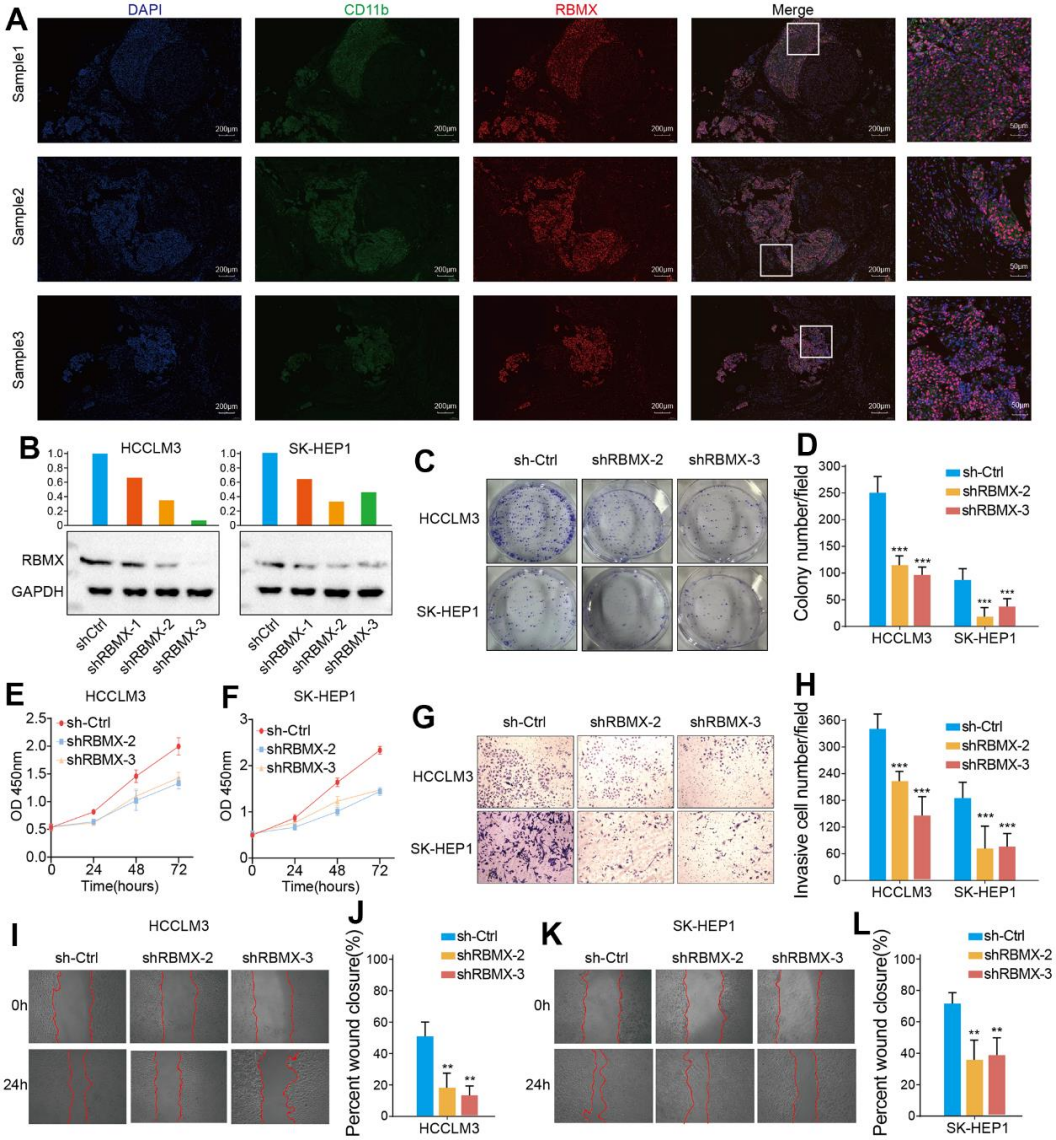
**TIMER immune cell infiltration analysis.** Heatmap illustrating the correlations of RBMX expression with the infiltration levels of CD4+ T cells, CAF, progenitor, Endo, Eos, HSC, Tfh, gdT, NKT, regulatory T cells (Tregs), B cells, neutrophils, monocytes, macrophages, dendritic cells, NK cells, mast cells, and CD8+ T cells in the pan-cancer setting. Red and blue squares indicate positive and negative correlations, respectively. CAF, cancer-associated fibroblasts; Endo, endothelial cells; Eos, eosinophils; gdT, γ/δT cells; HSC, hematopoietic stem cells; NK, natural killer; NKT natural killer T; RBMX, RNA binding motif protein X-linked; TIMER, Tumor IMmune Estimation Resource; Tfh, follicular helper T cells.

Analysis of immune cell infiltration in the pan-cancer setting was conducted to further assess the association between RBMX and cancer immunity using Spearman correlation analysis. Data were extracted from the Tumor IMmune Estimation Resource 2 (TIMER2) database. The results revealed the infiltration levels of CD4+ T cells, cancer-associated fibroblasts, lymphoid progenitor cells, myeloid progenitor cells, endothelial cells, eosinophils, hematopoietic stem cells, follicular helper T cells, γδ T cells, NK T cells, Treg cells, myeloid-derived suppressor cells (MDSC), neutrophils, monocytes, B cells, DC, macrophages, mast cells, NK cells, and CD8+ T cells in the pan-cancer setting. The infiltration levels of progenitor cells and MDSC were positively correlated with RBMX in most TCGA cancer types (Figure 5).

The infiltration level of MDSC with remarkably upregulated RBMX expression was high in ACC, LIHC, KIRP, and SKCM. In ACC, KRIP, and LIHC, RBMX was identified as a significant risk factor. These results suggested that overexpression of RBMX may promote tumor progression in ACC, LIHC, KIRP, and SKCM. MDSC are viewed as a heterogeneous population of myeloid cell precursors, immature granulocytes, monocytes, and DC, also termed immature immunosuppressor cells [22].

Previous studies suggested that MDSC can perform an immunosuppressive function in several types of cancer through a variety of pathways and mechanisms, thereby indirectly inhibiting cancer immune response [23]. Our results suggested that upregulated RBMX expression may affect the occurrence, prognosis, and treatment of cancer, and it is highly correlated with infiltrating MDSC. However, further investigation is warranted to determine the regulatory mechanism underlying this relationship.

### Correlations between RBMX and immune tumor mutation burden (TMB), microsatellite instability (MSI), and regulators

**Figure 6:**
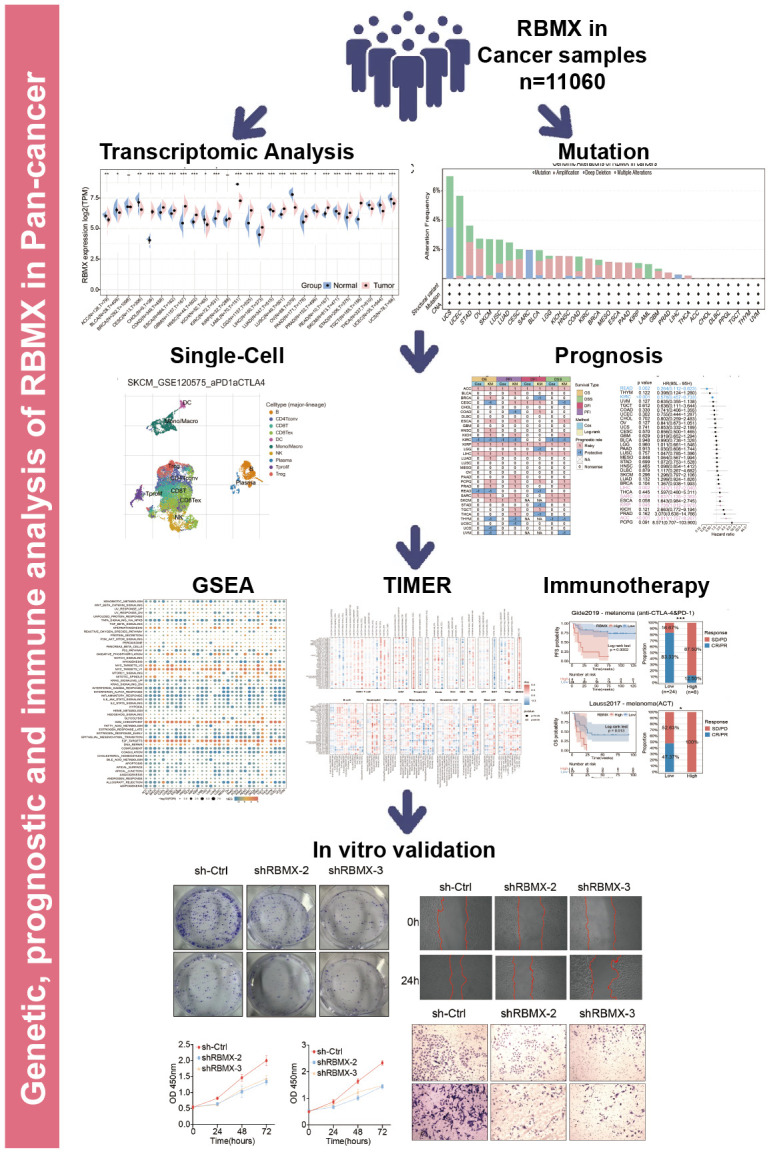
**Predictive role of RBMX and its relationships with immune regulators, TMB, and MSI.** (**A**) Correlations of RBMX expression with 47 types of immune regulators in the pan-cancer setting, illustrated by the Spearman correlation analysis. Red and blue squares represent positive and negative correlations, respectively. (**B**) Correlations between RBMX expression and MSI in the pan-cancer setting. (**C**) Correlations between RBMX expression and TMB in the pan-cancer setting. (**D**) Low- and high-RBMX subgroups distinguished based on the Kaplan–Meier curves in the Gide2019 cohort (anti-CTLA-4 and anti-PD-1, melanoma). Proportion of patients with melanoma who responded to anti-CTLA-4 and anti-PD-1 therapy in the low- and high-RBMX subgroups of the IMvigor210 cohort. (**E**) Low- and high-RBMX subgroups distinguished based on the Kaplan–Meier curves in the Riaz2017 cohort (anti-PD-1, melanoma). Proportion of patients with melanoma who responded to anti-PD-1 therapy in the low- and high-RBMX subgroups of the IMvigor210 cohort. (**F**) Low- and high-RBMX subgroups distinguished based on the Kaplan–Meier curves in the Lauss2017 cohort (ACT, melanoma). Proportion of patients with melanoma who responded to ACT therapy in the low- and high-RBMX subgroups of the IMvigor210 cohort. (**G**) Low- and high-RBMX subgroups distinguished based on the Kaplan–Meier curves in the Vanallen2015 cohort (anti-CTLA-4, melanoma). Proportion of patients with melanoma who responded to ACT therapy in the low- and high-RBMX subgroups of the IMvigor210 cohort. *p < 0.05, **p < 0.01, and ***p < 0.001 indicate statistical significance. ACT, adoptive cell transfer therapy; CTLA-4, cytotoxic T-lymphocyte associated protein 4; MSI, microsatellite instability; PD-1, programmed cell death-1; RBMX, RNA binding motif protein X-linked; TMB, tumor mutation burden.

The correlations between RBMX and immune regulators in the pan-cancer setting are illustrated in a heatmap (Figure 6A). The results showed remarkable positive relationships between RBMX and immune regulators in HNSC, KICH, LIHC, and pancreatic adenocarcinoma, as well as negative relationships in GBM, SARC, and testicular germ cell tumors (TGCT). Moreover, RBMX was significantly positively associated with CD200, CD276, and butyrophilin like 2 (BTNL2) expression in most types of cancer. According to these data, RBMX may act as an immune-related RNA-binding protein-coding gene in numerous types of cancer.

Consequently, we assessed the predictive role of RBMX in cancer patients treated with ICIs. We examined the correlations between RBMX expression and TMB, as well as MIS with deep insight across various types of cancer. RBMX was positively correlated with MSI in UCEC, TGCT, STAD, SARC, READ, and ESCA. It was also positively correlated with TMB in PRAD, LGG, LAML, HNSC, DLBC, and STAD. In contrast, RBMX was negatively correlated with TMB in THCA, THYM, THCA, KIRC, and BRCA (Figure 6B, 6C).

### RBMX predicted the efficacy of immunotherapy in the pan-cancer setting

We sought to further explore the predictive role of RBMX for the efficacy of immunotherapy in the pan-cancer setting. For this purpose, we utilized four cohorts of patients with melanoma who received ICI therapy. The results showed that the low-RBMX group had a better survival rate and time than the high-RBMX group. In the Gide2019 cohort, the response rate to anti-cytotoxic T-lymphocyte associated protein 4 (anti-CTLA-4) and anti-programmed cell death-1 (anti-PD-1) therapy was significantly higher in the low-RBMX group versus the high-RBMX group (83.33% vs. 12.50%, respectively) (Figure 6D). Moreover, Lauss et al. reported that the response rate to adoptive cell transfer therapy was 47.37% higher in the low-RBMX group (Figure 6E). Analysis of the other two cohorts revealed a similar trend; the low-RBMX group was associated with a higher OS probability (Figure 6F, 6G). The results indicated the important role of RBMX in predicting the efficacy of ICIs in the treatment of melanoma.

### RBMX correlated with CD11b+ MDSC infiltration in LIHC

**Figure 7:**
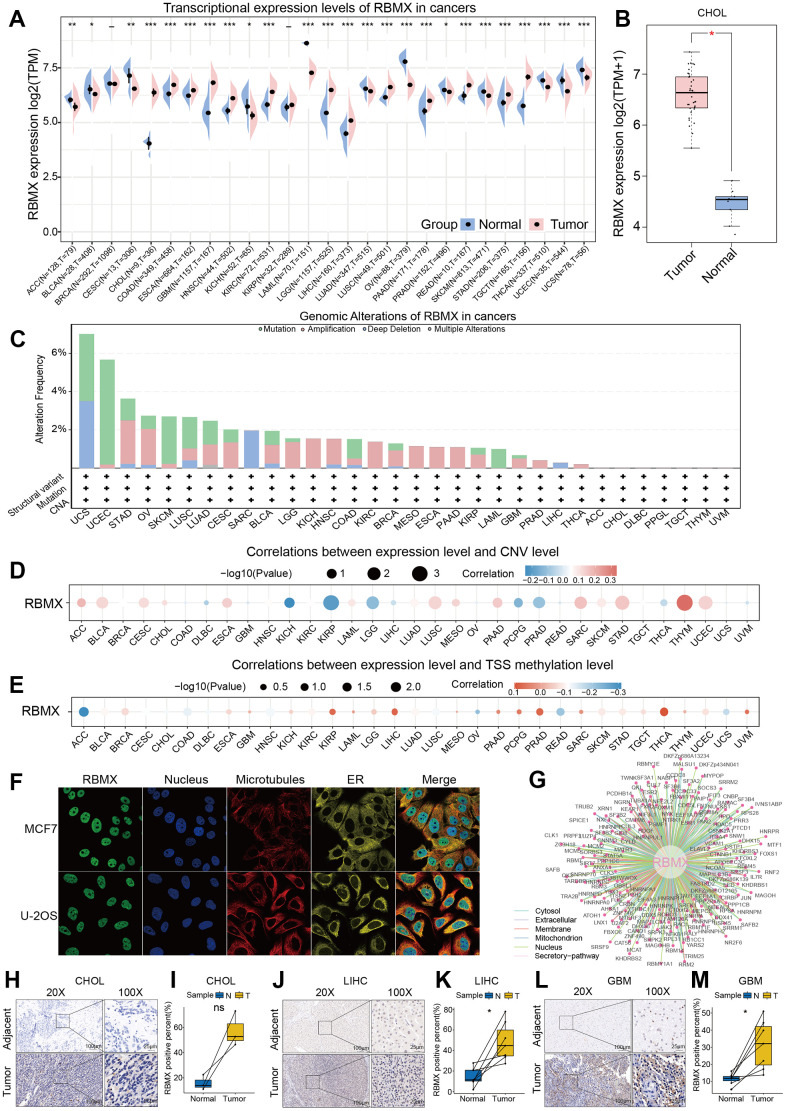
**Detection of CD11b+ cells and RBMX through fluorescence immunohistochemistry assay, and *in vitro* validation of the functions of RBMX in regulating the proliferative, migrative, and invasive abilities of LIHC cells.** (**A**) Co-fluorescence immunohistochemical analysis of RBMX and CD11b in tumor regions and adjacent normal tissues in three LIHC samples. (**B**) Western blots showing the RBMX knockdown effects of the three shRNAs in HCCLM3 and SK-HEP1 cells. (**C**, **D**) RBMX knockdown reduced the ability of HCCLM3 and SK-HEP1 cell lines for cell colony formation. (**E**, **F**) CCK8 assay also validated that RBMX knockdown can reduce the proliferation of HCCLM3 and SK-HEP1 cells. (**G**–**L**) Downregulation of RBMX also inhibited the invasive (**G**, **H**) and migratory (**I**–**L**) abilities of HCCLM3 and SK-HEP1 cells *in vitro*. CCK8, Cell Counting Kit-8; LIHC, liver hepatocellular carcinoma; RBMX, RNA binding motif protein X-linked; shRNAs, short hairpin RNAs.

CD11b is a biomarker of human MDSC [23]. We demonstrated that RBMX is overexpressed in LIHC samples, correlates with the prognosis of patients with LIHC, and is highly associated with MDSC infiltration in LIHC. Therefore, we conducted a fluorescence immunohistochemistry assay to detect RBMX and CD11b in three liver cancer samples. RBMX and CD11b were highly co-expressed in liver tumors compared with adjacent regions (Figure 7A). These results showed a potential relationship between RBMX and CD11b+ MDSC in liver cancer.

### RBMX regulated the proliferation, migration, and invasion of liver cancer cells *in vitro*

We also aimed to determine the potential biological functions of RBMX in liver cancer cells. Hence, we designed three shRNA plasmids to knock down the expression of RBMX in HCCLM3 and SK-HEP1 cell lines, and validated the efficiency of each shRNA in those cell lines (Figure 7B). We selected the shRBMX-2 and shRBMX-3 plasmids for subsequent cell function analysis because of their higher knockdown effects. Cell colony formation assays were performed using HCCLM3 and SK-HEP1 cell lines. The results showed that the ability of the two liver cancer cell lines for colony formation was significantly decreased following the knockdown of RBMX expression (Figure 7C, 7D). Consistent with these findings, the Cell Counting Kit-8 assays also revealed that the proliferative ability of HCCLM3 and SK-HEP1 cell was weakened by RBMX knockdown (Figure 7E, 7F). In addition, the results of Transwell invasion and wound-healing assays highlighted the prominent role of RBMX in regulating the mobility of LIHC cells. RBMX knockdown observably impaired the ability of HCCLM3 and SK-HEP1 cells to cross the Matrigel matrix in the Transwell system (Figure 7G, 7H), and inhibited cell mobility in *in vitro* two-dimensional culture (Figures 7I–7L).

## DISCUSSION

Cancer poses a major threat to modern society. Immunotherapy has emerged as a novel option for the treatment of cancer [24]. Nevertheless, research on immunotherapy is currently hampered by the need to overcome the occurrence of adverse events related to the regulation of the immune system, including autoimmunity and nonspecific inflammation. However, despite its effectiveness in the pan-cancer setting, only a small proportion of patients can benefit from checkpoint blockade [25]. Therefore, additional research on the immune checkpoint may improve the clinical therapy of cancer [26]. In a recent study, RBMX (acting as a protein connector) was strongly correlated with T-cell lymphoma progression and chemotherapy response [27]. Nonetheless, it has also been identified as a potential suppressive factor in lung cancer and oral squamous carcinoma [9, 28–30]. In the present study, we performed a comprehensive analysis of RBMX in the pan-cancer setting, and verified its effective role in the prediction of immunotherapy response. Furthermore, our results provided clues for further investigating the involvement of RBMX in cancer progression and immunotherapy.

Initially, we extracted data from TCGA and GTEx databases to detect differences in the expression levels of RBMX between tumor and normal tissues. The analysis revealed that RBMX is highly expressed in most cancer types. CNV level analysis indicated a remarkable correlation between RBMX and THYM, while TSS methylation level analysis showed a significant correlation between RBMX and TSS methylation in THCA. These results revealed a potential mechanism through which the upregulation of RBMX expression affects tumor progression by influencing RNA homeostasis.

Next, we conducted single-cell analysis for the expression of RBMX in the pan-cancer setting. The data demonstrated that RBMX was highly expressed in immune cells and plasma. Analysis of the glioma microenvironment indicated high expression of RBMX in malignant cells and monocytes/macrophages. Previous studies showed that RBMX plays an essential role in the progression of LUAD through combination with miR-19b-3p [31]. According to our results, may RBMX mediate the immune microenvironment by binding to certain RNA.

Subsequently, we analyzed the prognostic value of RBMX in the pan-cancer setting. For this purpose, we assessed the OS, PFI, DFI, and DSS. The results consistently showed that RBMX was closely associated with the prognosis of cancer. Previous research verified that low RBMX expression is related to poor prognosis in endometrial cancer [32, 33] and bladder cancer [34]. However, high expression of RBMX has also been associated with poor prognosis in hepatocellular carcinoma (HCC) [35]. The conclusions of the present study are consistent with those of previous investigations.

Next, we performed GSEA of RBMX, which demonstrated a significant correlation between RBMX expression and immune-related pathways, in particular TNFA signaling via NFKB, IFNG response, IFNA response, and inflammation. These results prompted us to investigate the correlations between RBMX and response to cancer immunotherapy.

Moreover, immune cell infiltration analysis revealed that the infiltration levels of progenitor cells and MDSC were positively related to RBMX expression in most cancer types. MDSC acted as protectors of cancer, preventing the exposure of cancer cells to the immune system and enhancing resistance to immunotherapy [36]. Research studies showed that MDSC suppressed the function of NK cells in cancer cell infiltration of the immunosuppressive tumor microenvironment [37]. Our findings emphasized the correlations between RBMX and MDSC in the tumor microenvironment, as well as the role of RBMX as a predictor of response to immunotherapy. Based on this evidence, RBMX might influence the infiltration of MDSC to promote tumor progression and suppress the sensitivity of cancer cells to ICI therapy.

Intriguingly, RBMX was significantly linked to immune regulators and positively associated with CD200, CD276, and BTNL2. Subsequently, we confirmed the robust predictive role of RBMX in terms of outcome of ICI therapy. We evaluated the predictive role of RBMX in four cohorts of patients with cancer who received immunotherapy. The results showed that patients expressing low levels of RBMX had better survival rate and time than those expressing high levels of RBMX. This finding is consistent with the hypothesis that RBMX may affect the tumor suppressive immune microenvironment by regulating MDSC infiltration through an unrevealed mechanism.

Admittedly, the present study was characterized by several limitations. The pan-cancer research data were obtained from online open databases. Currently, there is a lack of large-scale clinical cohort data to support the present conclusions. Moreover, further experiments should be conducted to identify the specific mechanism underlying the regulatory effect of RBMX on tumor progression, particularly in terms of regulating the proliferation, migration, and invasion of cancer cells.

In conclusion, we performed an integrated analysis of RBMX, revealing its effective role in predicting cancer prognosis and response to immunotherapy. Abnormal expression of RBMX is associated with immune regulation, prognosis, the tumor microenvironment, immune cell infiltration, MSI, and TMB. The results of this study indicated that RBMX may play an independent role in clinical diagnosis and prediction. In the future, targeting of RBMX may be a novel method in cancer therapy.

## Supplementary Material

Supplementary Table 1

Supplementary Table 2
